# Miniaturized Vis–NIR handheld spectrometer for non-invasive pigment quantification in agritech applications

**DOI:** 10.1038/s41598-023-36220-2

**Published:** 2023-06-12

**Authors:** U. S. Dinish, Mark Teo Ju Teng, Valerie Teo Xinhui, Kapil Dev, Javier Jingheng Tan, Sally Shuxian Koh, Daisuke Urano, Malini Olivo

**Affiliations:** 1 Institute of Materials Research and Engineering (IMRE), Agency for Science, Technology and Research (A*STAR), 2 Fusionopolis Way, Innovis #08-03, Singapore, 138634 Singapore; 2 Institute of Bioengineering and Bioimaging, Agency for Science, Technology and Research (A*STAR), Singapore, Singapore; 3 Temasek Life Sciences Laboratory, National University of Singapore, Singapore, Singapore; 4 Department of Biological Sciences, National University of Singapore, Singapore, Singapore

**Keywords:** Plant sciences, Energy science and technology, Engineering, Optics and photonics

## Abstract

Advanced precision agriculture requires the objective measurement of the structural and functional properties of plants. Biochemical profiles in leaves can differ depending on plant growing conditions. By quantitatively detecting these changes, farm production processes can be optimized to achieve high-yield, high-quality, and nutrient dense agricultural products. To enable the rapid and non-destructive detection on site, this study demonstrates the development of a new custom-designed portable handheld Vis–NIR spectrometer that collects leaf reflectance spectra, wirelessly transfers the spectral data through Bluetooth, and provides both raw spectral data and processed information. The spectrometer has two preprogramed methods: anthocyanin and chlorophyll quantification. Anthocyanin content of red and green lettuce estimated with the new spectrometer showed an excellent correlation coefficient of 0.84 with those determined by a destructive gold standard biochemical method. The differences in chlorophyll content were measured using leaf senescence as a case study. Chlorophyll Index calculated with the handheld spectrometer gradually decreased with leaf age as chlorophyll degrades during the process of senescence. The estimated chlorophyll values were highly correlated with those obtained from a commercial fluorescence-based chlorophyll meter with a correlation coefficient of 0.77. The developed portable handheld Vis–NIR spectrometer could be a simple, cost-effective, and easy to operate tool that can be used to non-invasively monitor plant pigment and nutrient content efficiently.

## Introduction

Leaf colour pattern varies depending on age^1–3^, pathogen infection^4–6^, environmental^7,8^, and nutritional stresses^9^. Thus, these patterns are widely used to diagnose plant health statuses in agricultural fields. Vis–NIR handheld sensor measures the spectral pattern of leaf reflectance from ∼400 to ∼900 nm that cover absorption peaks of all phytopigments. With a higher resolution in wavelength space, the spectral information allows us to accurately estimate the concentration of chlorophyll, anthocyanin, and polyphenols in a non-destructive manner^10^. Most handheld sensors specialized for plant diagnostics provide limited information of individual plant constituents such as anthocyanin and chlorophyll while general handheld spectrometers measure and save raw reflectance intensities from which users need to extract the information of interest.

Investigating anthocyanin content is a continuing interest within the agriculture research community due to its high antioxidant properties, which are known for myriad of health benefits^11^. Consumption of anthocyanin rich foods has been shown to reduce the risk of cardiovascular disease^12^, type 2 diabetes^13^, and inflammation^14^. In plants, the amount of anthocyanin determines the color and appearance of the leaves, which serve as one of the markers of quality^15^. Currently, the conventional form of anthocyanin measurement utilizes either chemical or biochemical techniques. The chemical approach involves harvesting the leaves followed by multiple extraction steps^16^. Such a method is thus, time-consuming, and expensive. Another quantifying method is biochemical processes where leaves are analyzed using one of the following techniques: gas chromatography (GC), high performance liquid chromatography (HPLC), gas chromatography-mass spectrometry (GC–MS), liquid chromatography-mass spectrometry (LC–MS) etc.^17^. The aforementioned techniques are laborious, expensive, require special equipment, and regrettably destructive.

Optical spectroscopy is a rapid, non-destructive analytical technique that uses the concept of light reflectance to evaluate leaf structures and their biochemical properties. The intensity of the reflected light at various wavelengths depends on the absorption of light by the pigments present in the leaf. This allows for the quantitative measurement of the functional properties of plants such as carotenoids and anthocyanin. Optical spectroscopy has been widely adopted for plant health monitoring^18–20^. Presently, several expensive industrial systems that measure leaf pigments are available, such as the SpectraVue Leaf Spectrometer^21^ and the SPAD 502 Plus Chlorophyll Meter^22^. There are also many hyperspectral setups using robust analytical methods like Partial Least Square Regression (PLSR) to estimate leaf properties^23–25^. However, these systems are either bulky or limited in the type of pigments detectable. Considering the above, we have developed a cost effective, portable, and easy to use handheld Vis–NIR spectrometer to quantify the plant pigments in a non-invasive manner. The handheld spectrometer is relatively smaller in size and comparable in weight with respect to the abovementioned commercial systems. Unlike most commercial systems, the handheld spectrometer acquires measurement without clamping the leaf sample preventing micro damage to the sample.

The capability of the spectrometer is also expandable to include chlorophyll quantification, which is crucial in determining senescence in plants. Unlike senescence in animal cells, where the cell cycle arrest in damaged or aged cells^26^, plant senescence refers to the essential redistribution of nutrients to ensure species propagation^27,28^. Leaf yellowing is usually the tell-tale sign of chlorophyll degradation, which indicates the initiation of senescence. Yet even before yellowing becomes visible, chlorophyll levels have already started to decrease^10,29^. Therefore, the timing of crop harvest is pivotal as premature senescence can reduce final crop yield while overdue harvesting can compromise nutrient quality of the crop^30,31^.

In this study, we have newly designed and developed an affordable handheld device that collects leaf reflectance spectra, wirelessly transfers the spectral data through Bluetooth, and provides both raw spectral data and processed information (estimated chlorophyll and anthocyanin contents) to users. We begin by explaining the intuition behind the design and construction of the spectrometer. Next, using anthocyanin and chlorophyll content as case studies, we successfully showed that our device is capable of accurately quantifying these phytopigments. Data obtained from the spectrometer significantly correlated with existing conventional established methods of quantitating anthocyanins and chlorophyll. These findings strongly suggest that our developed portable handheld Vis–NIR system has utility as a non-invasive and cost-effective method of monitoring plant pigment levels.

## Methods

### Handheld spectrometer system integration

**Figure 1:**
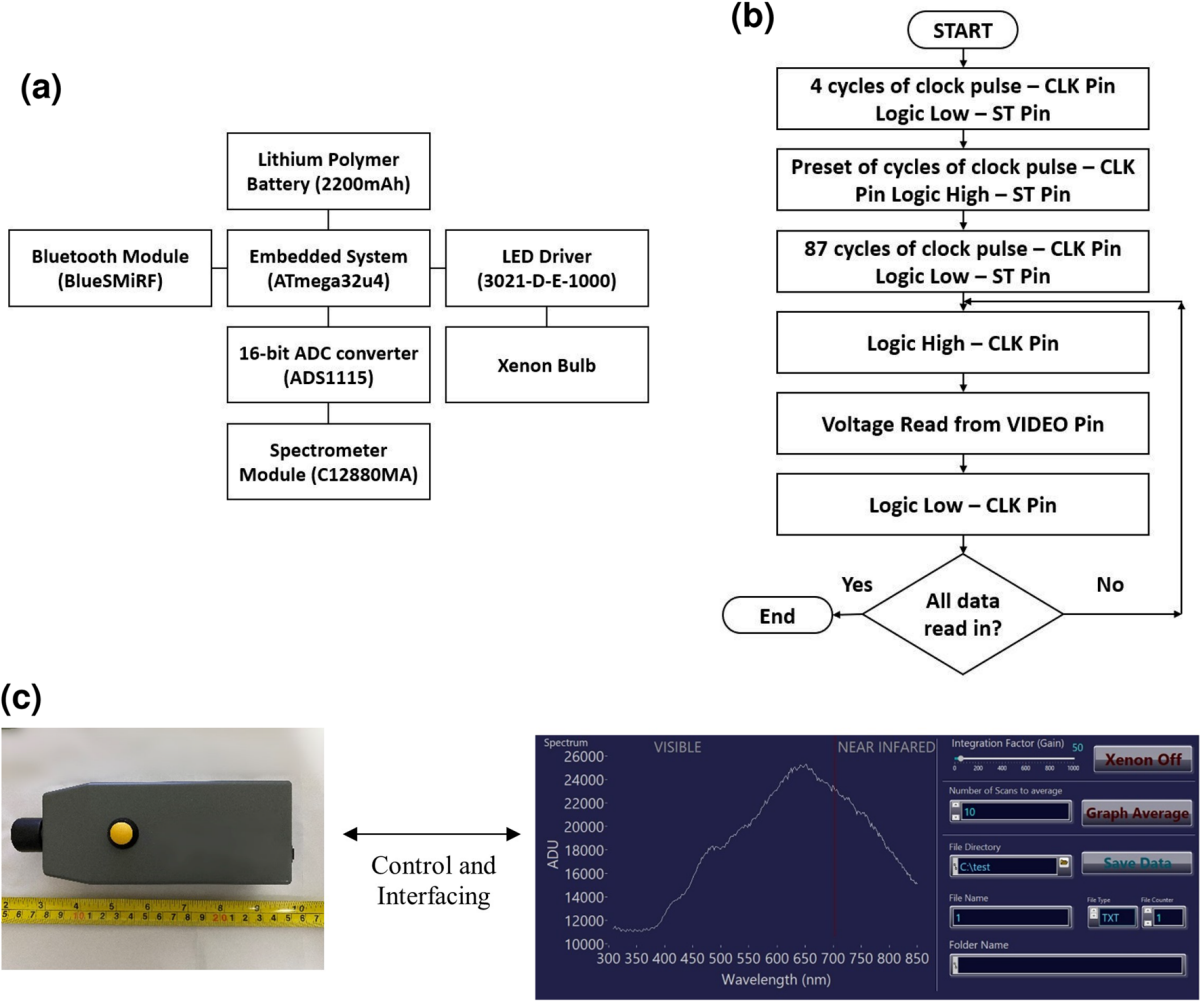
Handheld spectrometer system integration. (**a**) Block diagram of the system setup. (**b**) Flow chart describing the flow to read data from C12880MA. (**c**) Complete setup of the handheld spectrometer system.

The construction of the spectrometer system involves the integration of hardware, firmware, and software as shown in Fig. 1.

Figure 1a describes how the individual electronic components are linked to one another. The embedded system uses Adafruit Itsy Bitsy microcontroller board based on the ATmega32u4 chipset powered by a lithium polymer battery for portability. An external Bluetooth module was used to interface with the embedded system to enable data transfer out of the device wirelessly. A 16-bit Analog-to-Digital Converter (ADC) was used to improve the resolution of the data from the spectrometer module. An LED Driver was used to ensure the current supplied to the Xenon bulb was properly regulated for consistent brightness which was critical to the final read-out data of the Hamamatsu C12880MA spectrometer module^32^.

Figure 1b shows the flow of data acquisition from the spectrometer module. The firmware was developed in Arduino Integrated Development Environment (IDE) version 1.8.19. The definition of a clock pulse cycle consists of logic-high (5 V) and logic-low (0 V) with a one millisecond delay for both clock-high and clock-low periods. In the case of the spectrometer, 4 initial cycles of clock pulses were introduced to the CLK (clock) pin of the spectrometer while the ST (start) pin was kept at logic-high. This was followed by introducing a logic-high to the ST pin while the CLK pin was fed with the number of clock pulses, which is directly related to the gain of the readout from the spectrometer module. Finally, a logic-low is introduced to the ST pin while the CLK pin received 87 cycles of clock pulses allowing the data available for acquisition to be found at the VIDEO pin.

To successfully acquire the data, the CLK pin was introduced a logic-high before the voltage at the VIDEO pin is read into the microcontroller via 16-bit ADC. Hereinafter, a logic-low was implemented to the CLK pin. This process was repeated 288 times to read all the amplitudes of their corresponding wavelength^33^ between 350 and 850 nm. The interfacing software was developed using LabVIEW Full Development System for the user to visualize data from the handheld spectrometer. Figure 1c shows the interface that users can utilize to save data to the local computer drive, switch on/off the xenon bulb, and perform real-time averages for a stable spectrum display. The final dimension and weight of the handheld spectrometer are 150.0 mm $\times$ 55.0 mm $\times$ 58.0 mm and ∼200 g respectively. The total cost of the prototype version of the handheld spectrometer is estimated to be USD 1000.

We validate the efficacy of this portable handheld Vis–NIR spectrometer for routine analysis of plant stress and nutrients using two case studies involving the estimation of plant pigments, anthocyanin and chlorophyll, under different conditions.

### Anthocyanin quantification

In the first experiment, a total of 60 Batavia lettuce (30 green and 30 red) were chosen to validate the capability of the spectrometer in quantifying the anthocyanin levels. For each leaf, measurements were taken 2 cm above the surface of the leaf using an attached spacer. At each surface, the measurement was repeated thrice with the averaged value used for further analysis. From the acquired spectra, Modified Anthocyanin Reflectance Index (mARI), a well-established vegetation index^34,35^ as shown in Eq. 1, was used to quantify the amount of anthocyanin non-invasively in each leaf.

1 $$\mathrm{mARI}= \frac{1}{550\mathrm{nm}}- \frac{1}{700\mathrm{nm}}$$

The leaves were measured for fresh weight and snap frozen in liquid nitrogen until biochemical processing was performed^36^. Subsequently, the mARI value was correlated to the anthocyanin amount obtained from conventional biochemical analysis. Eight spectra were removed from the correlation analysis as the size of those leaves were too large to carry out the standard biochemical analysis. A total of 26 green and 26 red leaves were used for correlation analyses. Correlation was evaluated using Pearson’s coefficient along with the p-value significance, R-squared value, and root mean square error.

### Chlorophyll quantification

**Figure 2:**
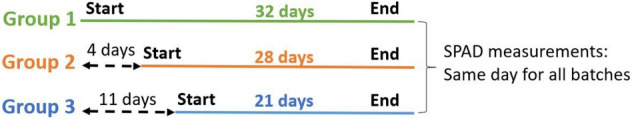
Schematic showing the plant germination and growth timeline for the three different groups of plants. Plants in Group 1 were germinated the earliest. Group 2 started 4 days after Group 1, while Group 3 started 11 days after Group 1. The desired plant ages of 21, 28, and 32 days were attained at the end of the growth period. Soil–plant analysis development (SPAD) measurements were then taken on the same day for all three batches.

In the second experiment, a longitudinal experiment for evaluating plant senescence was conducted. Plant senescence was used as a model to evaluate chlorophyll content in the leaves to validate the efficacy of the systems. Seeds (Lactuca sativa var. Little Gem) were surface sterilized with a 10% bleach solution containing Triton X-100. Following which, these seeds were plated onto on Murashige and Skoog (MS) basal media with 1% agar. Seeds were left to germinate and grow in a growth chamber at 22 °C under 24 h continuous light and ∼90 µmolm^−2^* s*^−1^ light intensity. After four days, uniformly sized lettuce seedlings were selected and transferred to a soil mixture composed of BVB substrates (Kekkilä-BVB, Netherlands) and sand in a 10:1 ratio. For the remaining growth period, plants were grown at 22 °C under a 16 h light/8 h dark cycle, ∼150 µmolm^−2^ s^−1^ light intensity, and 70% relative humidity. In three separate groups, 10 plants in each group were grown to 21-, 28-, and 32-days-old respectively, according to Fig. 2. Plants were kept well-watered throughout the growth period. Plant germination was scheduled such that all groups of plants were measured for chlorophyll content on the same day.

Spectral measurements were taken thrice on the 3rd and 4th leaves of the 10 plants in each group using the handheld spectrometer. These measurements were averaged for subsequent analysis. Merris Terrestrial Chlorophyll Index (MTCI) is a conventional metric used to determine chlorophyll content in plants^37–39^. To compare the changes in chlorophyll content between older and younger plants, MTCI is calculated using Eq. 2 from the acquired spectra.

2 $$\mathrm{MTCI}= \frac{754\mathrm{nm}-709\mathrm{nm}}{709\mathrm{nm}-681\mathrm{nm}}$$

Subsequently, the MTCI values obtained from the handheld Vis–NIR spectrometer were correlated with the chlorophyll content measured non-destructively using a portable commercial chlorophyll meter (SPAD-502, Konica Minolta, Japan) to obtain the Pearson’s coefficient, p-value significance, R-squared value, and root mean square error.

## Results and discussion

### Characterization of spectrometer

**Figure 3:**
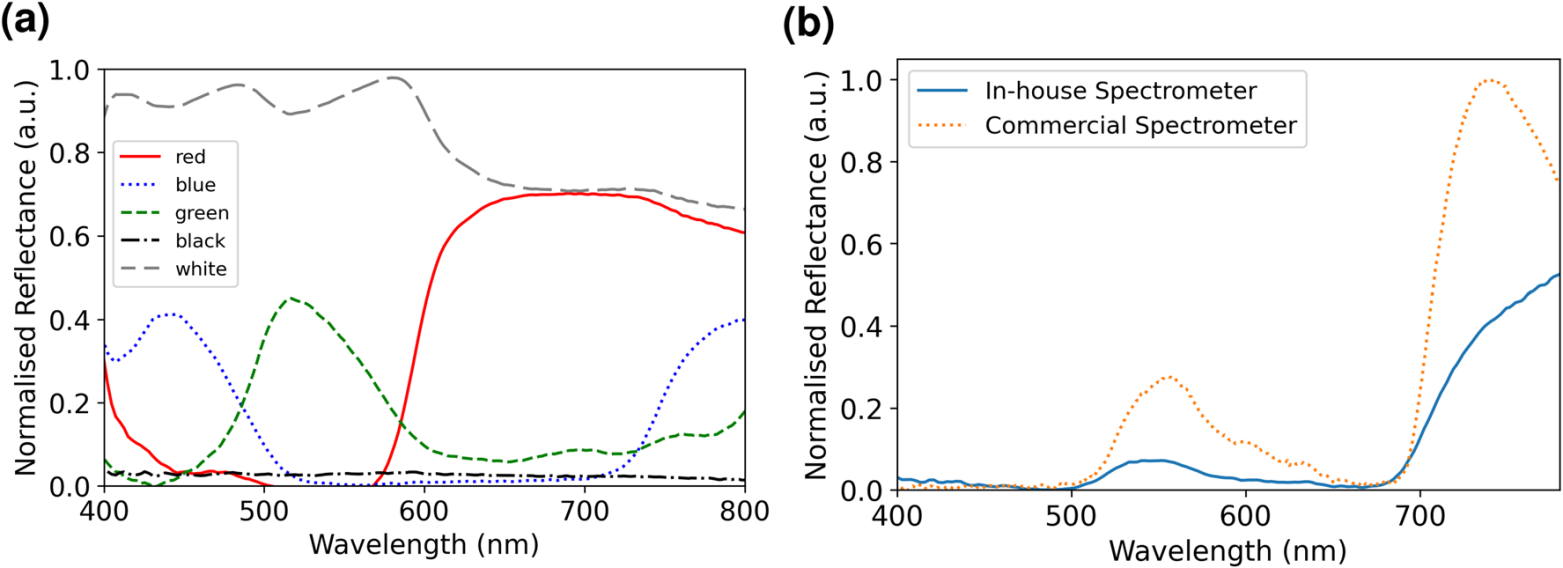
Characterization of handheld spectrometer. (**a**) Color characterization with ‘Colorchecker classic’ standardized target for red, blue, green, black and, white. (**b**) Comparison of spectra acquired from portable handheld Vis–NIR spectrometer with commercial Arcoptic Vis–NIR-FIB fibered spectrometer on Green Lettuce.

A color characterization test was done with a standardized color target (Colorchecker classic) from X-Rite, Inc. Figure 3a shows the normalized reflectance obtained from the color characterization test. From this test, we note that the spectrometer can correctly detect the wavelength range of the primary colors of light while absorbing all the light in black color and reflecting almost all the light with white. In addition to the color characterization test, the portable handheld Vis–NIR spectrometer was also compared with a fiber probe based commercial spectrometer (ARCoptix VIS–NIR-FIB). From Fig. 3b, it is observed that the measured spectral peaks of green lettuce from the portable handheld Vis–NIR spectrometer are identical to that of the commercial spectrometer. However, we note that the handheld spectrometer exhibits spectral differences after 700 nm due to its lower quantum efficiency of the detector at higher wavelengths. Also, the ARCoptix spectral measurements were performed with a fiber-optic probe in contact with the leaf, whereas the developed handheld device operates in free-space mode measuring the reflectance at around 2 cm away from the sample.

### Anthocyanin quantification

**Figure 4:**
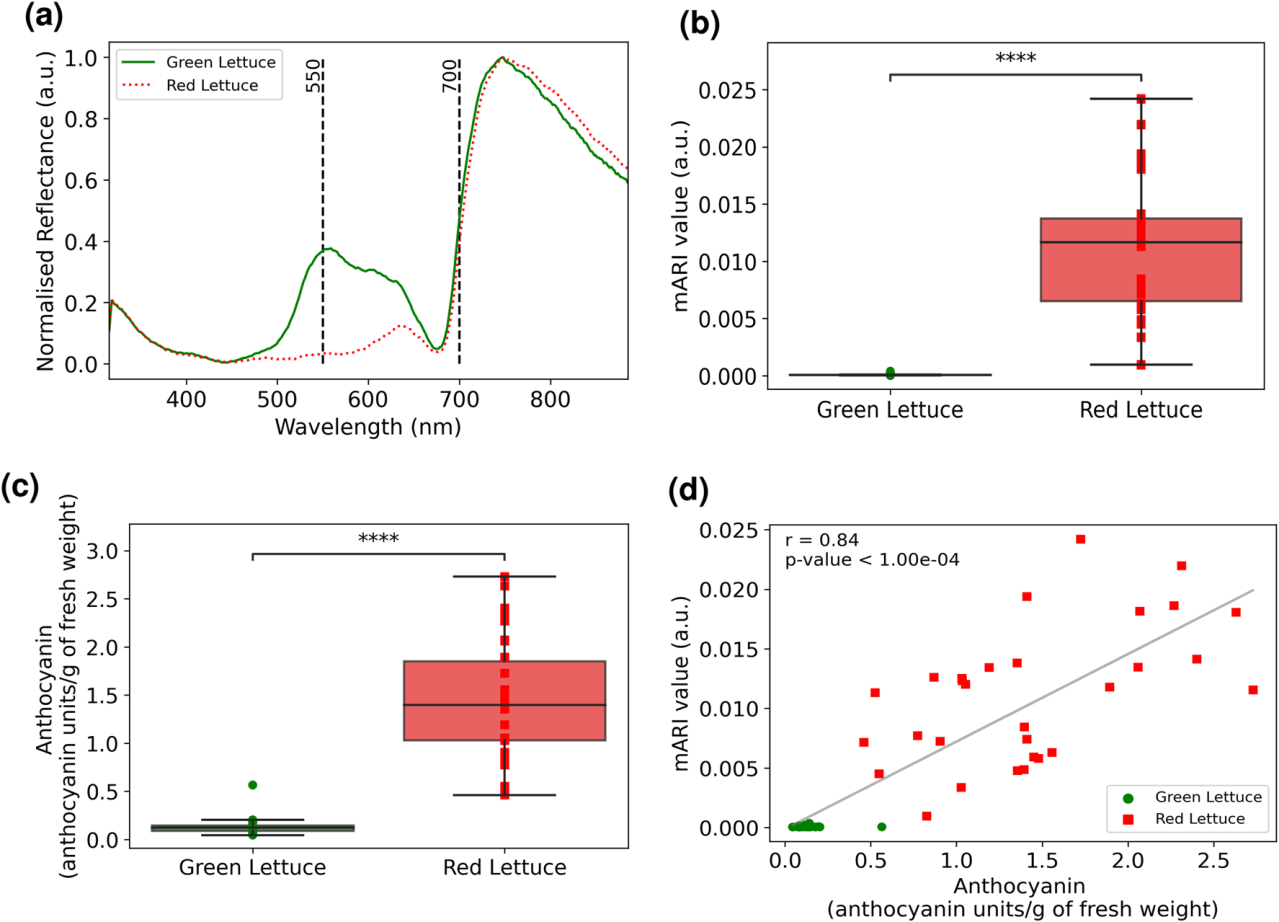
Anthocyanin quantification using handheld spectrometer. (**a**) Average reflectance spectra of red and green lettuce. Two vertical dotted lines show leaf reflectance at 550 nm and 700 nm which are wavelengths used for mARI calculations. (**b**) mARI values of green and red lettuce after outlier removal. From bottom up, the boxplot shows the minimum mARI values (lower whiskers), lower quartile (Q1), median (Q2), upper quartile (Q3), and maximum mARI values (upper whiskers). Two-sided Mann–Whitney-Wilcoxon test was performed yielding p-value of annotation legend: ****: p <  = 1.00 $\times$ 10^−4^. (**c**) Biochemical anthocyanin values of green and red lettuce after outlier removal. From bottom up, the boxplot shows the minimum anthocyanin values (lower whiskers), lower quartile (Q1), median (Q2), upper quartile (Q3), and maximum anthocyanin values (upper whiskers). Two-sided Mann–Whitney-Wilcoxon test was performed yielding p-value of annotation legend similar to (**b**). (**d**) Correlation between mARI index obtained using portable handheld Vis–NIR spectrometer against the anthocyanin contents quantitated biochemically to obtain a p-value of 4*.*70*X*10^−16^ with linear equation of y = 0.01x-0.00, *r*^2^ of 0.71, and RMSE of 1.14.

Figure 4a shows the first set of analyses comparing the mean spectral differences between green and red Batavia leaves. The difference in peak location for green and red leaves within the visible wavelength range is as expected for the two different colors. It agrees with the theory that green leaf contains green pigments which reflect light in the wavelength range of 520–560 nm while red pigments in red leaves reflect light in the wavelength range of 635–700 nm..

From Fig. 4b, red lettuce had significantly higher median mARI value of 0.013 compared to green lettuce (p < 0.0001) which indicates greater anthocyanin content in red lettuce. The same trend is also observed in the anthocyanin values obtained from the gold standard biochemical analysis with red lettuce having 1.25 anthocyanin units/g of fresh weight more anthocyanin than in green lettuce as shown in Fig. 4c.

Next, the mARI values were correlated with the gold-standard biochemical measurement. The results, as shown in Fig. 4d, indicates a strong positive correlation of 0.84, p-value significance of 4.70X10^−16^, R-squared value of 0.71, and root mean square error of 1.14. This result is highly promising as it indicates that our handheld spectrometer is capable of accurately detecting the amount of anthocyanin in leaves. Furthermore, the high correlation with the biochemically obtained anthocyanin values indicates that the spectrometer has the potential to be comparable with conventional biochemical analyses.

### Chlorophyll quantification

**Figure 5:**
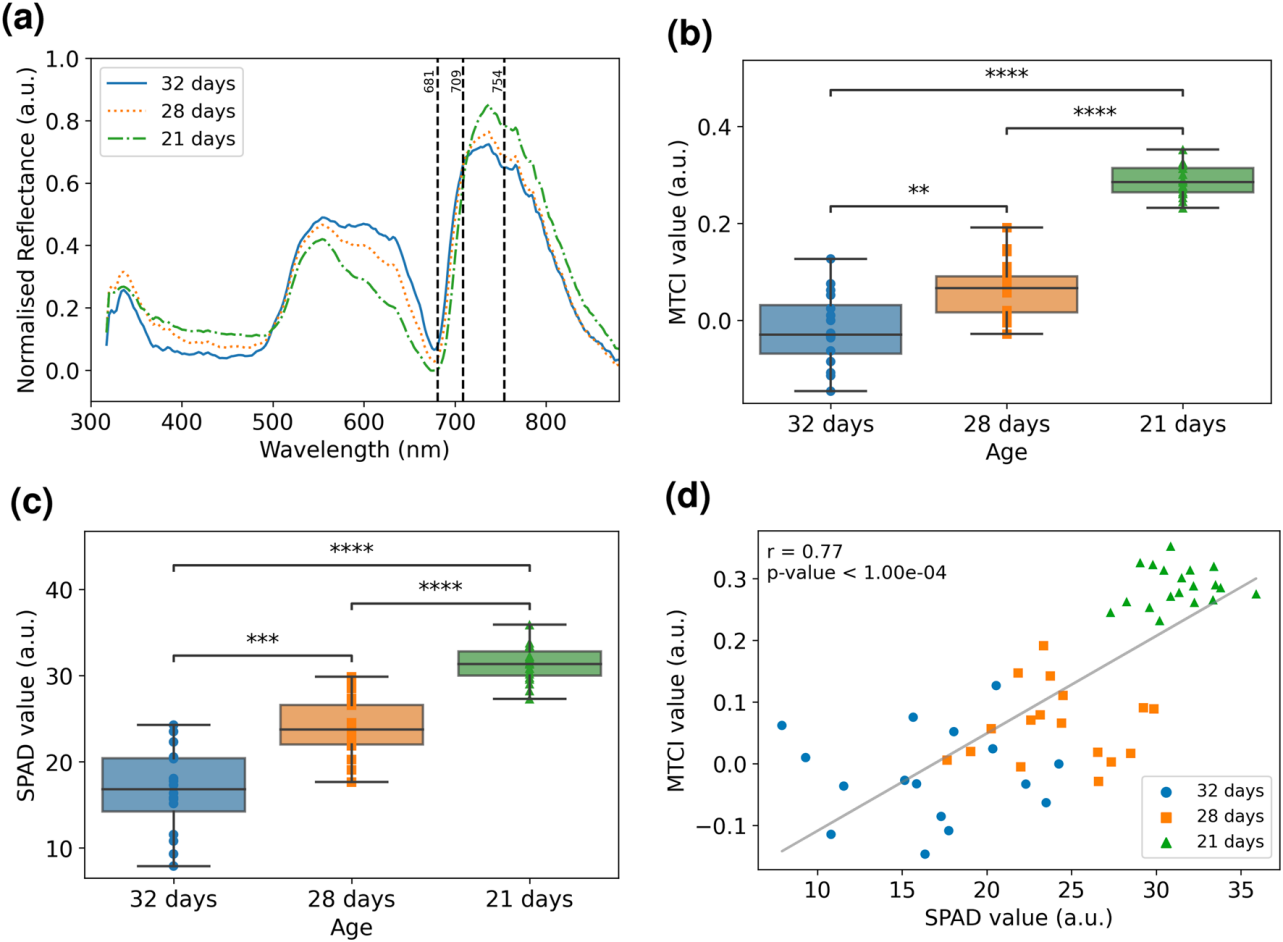
Chlorophyll quantification using handheld spectrometer. (**a**) Mean reflectance spectra of leaves of various age groups measured using handheld spectrometer. The blue, orange and green curves show the average spectra of each group. The vertical dotted lines indicate the corresponding wavelengths used for MTCI calculations. (**b**) MTCI values of leaves of various age groups after outlier removal. From bottom up, the boxplot shows the minimum MTCI values (lower whiskers), lower quartile (Q1), median (Q2), upper quartile (Q3), and maximum MTCI values (upper whiskers). Two-sided Mann–Whitney-Wilcoxon test was performed yielding p-value of annotation legend: **: 1.00X10^−3^ < p <  = 1.00X10^−2^; ***: 1.00X10^−4^ < p <  = 1.00X10^−3^; ****: p <  = 1.00X10^−4^. (**c**) SPAD values of leaves of various age groups obtained using commercial chlorophyll meter. From bottom up, the boxplot shows the minimum SPAD values (lower whiskers), lower quartile (Q1), median (Q2), upper quartile (Q3), and maximum SPAD values (upper whiskers). Two-sided Mann–Whitney–Wilcoxon test was performed yielding p-value of annotation legend similar to (**b**). (**d**) Correlation between MTCI index obtained using in-house Vis–NIR spectrometer against the SPAD value obtained with commercial system to obtain a p-value of 2*.*41*X*10^−11^ with linear equation of y = 0.02x−0.27, *r*^2^ of 0.59, and RMSE of 25.30.

The purpose of the experiment is to explore if the portable handheld spectrometer can detect chlorophyll degradation in plants of various ages, which is indicative of senescence. Figure 5a shows that the mean reflectance within the visible wavelength region (500 nm -700 nm) increases as the plant ages. In the near-infrared region (> 700 nm), there is a trend of decreasing mean reflectance with plant age. The process of age-induced senescence is known to reduce the chlorophyll content in plants^40^. This trend agrees with the work of Gitelson et al. (1996)^41^ that the reflectance in red edge decreases with leaf chlorophyll content.

From Fig. 5b, 21-day-old plants had the highest median MTCI value of 0.29, which were significantly higher compared to 28-day-old plants (p < 0.0001) and 32-day-old plants (p < 0.0001). 28-day-old plants had MTCI values of 0.07, which was significantly higher than 32-day-old plants (p < 0.001). Similar trend was also observed in the SPAD values obtained from a commercial spectrometer where 21-day-old plants had the highest median SPAD value followed by 28-day-old plants as shown in Fig. 5c. Together, these results show an age-dependent difference in MTCI values across treatment groups.

The observed differences in chlorophyll content follows the trend of age dependent senescence, whereby leaves lose chlorophyll in a sequential manner for the remobilization of nutrients. Next, as shown in Fig. 5d, the MTCI values were correlated with the chlorophyll values obtained from the commercial system to achieve a Pearson’s coefficient of 0.77, p-value significance of 2.41X10^−11^, R-squared value of 0.59, and root mean square error of 25.30, highlighting the capability of the spectrometer to be on par with the existing commercial system.

## Conclusion

This study sets out to validate the capability of the developed portable handheld Vis–NIR spectrometer to detect essential pigments non-invasively in plants. Through the two key experimental case studies involving anthocyanin and chlorophyll quantification, the performance of the spectrometer has proven to be comparable with existing established method/system. Our handheld spectrometer can detect anthocyanin content in plants with 0.84 correlation to conventional biochemical analyses. This is impressive as such high correlation was achieved using a non-invasive and portable device. The chlorophyll quantification experiment established that portable handheld spectrometer was able to differentiate changes in chlorophyll content effectively in plants of different ages. Moreover, the MTCI index derived from the portable handheld Vis–NIR spectrometer was able to achieve a strong correlation of 0.77 with the commercial SPAD system. The reliability of the system in chlorophyll quantification shows great utility in studying senescence in plants, which demonstrates its feasibility as a non-invasive plant health monitoring device. Hence, we envision that the developed Vis–NIR spectrometer can potentially be used in routine plant health monitoring under both indoor and outdoor farm settings. The developed system could also be used to monitor nutrient contents, evaluate plant stress, and ripeness of fruits. Further systemic improvements could incorporate a wider wavelength detector spectrometer to examine a variety of vegetation indices to quantify micronutrients, other phytopigments, and water content non-invasively. We are also currently in the process of validating the handheld spectrometer in outdoor field conditions where the device is subjected to environmental fluctuation. This will ensure that the device is stable and reproducible as with existing commercial systems.
